# Health Action Process Approach (HAPA) as a Framework to Understand Compliance Issues With Health Protocols Among People Undergoing Isolation at Emergency Hospital for COVID-19 Wisma Atlet Kemayoran and RSCM Kiara Ultimate Jakarta Indonesia

**DOI:** 10.3389/fpsyt.2022.871448

**Published:** 2022-05-26

**Authors:** Khamelia Malik, Nurmiati Amir, A. A. A. A. Kusumawardhani, Petrin Redayani Lukman, Rhapsody Karnovinanda, Leslie Melisa, Lidya Purnama Dewi, Agnes Lasmono

**Affiliations:** ^1^Department of Psychiatry, Faculty of Medicine, Cipto Mangunkusumo National Referral Hospital, Universitas Indonesia, Jakarta, Indonesia; ^2^Neuroscience and Brain Development Research Cluster, Faculty of Medicine, Indonesia Medical Education and Research Institute, Universitas Indonesia, Jakarta, Indonesia

**Keywords:** Health Action Process Approach, COVID-19, compliance with health protocols, isolation in health facility, Indonesia

## Abstract

**Introduction:**

This study aims to identify the psychosocial determinants and examine the mediation mechanisms of the compliance with COVID-19 health protocols among people undergoing isolation in health facilities that specifically treat COVID-19 cases in Jakarta, Indonesia.

**Methods:**

This is a cross-sectional study which used socio-cognitive approach, known as the Health Action Process Approach (HAPA), to understand the complexity of issues related to compliance with health protocols. A total of 1,584 subjects participated in this study, including 865 men and 719 women over the age of 18 years old during the data collection period (October 19–26, 2020). The data were collected using questionnaire that was developed by a team of experts from the Faculty of Medicine, Universitas Indonesia—Dr. Cipto Mangunkusumo General Hospital, and survivors. The data that has been collected were then analyzed using Structural Equation Modeling, a multivariate data analysis technique.

**Results:**

The final research model in this study fulfills the criteria for a good model fit. This study found that individuals who have strong self-efficacy regarding their ability to implement behaviors and overcome obstacles will have stronger intent to comply in the future. The study also found that stronger intent will lead to stronger planning, and planning was found mediating intention and compliance with health protocols.

**Conclusion:**

This research model is comprehensive and useful in understanding compliance with health protocols among people undergoing isolation in health facilities for COVID-19 (Wisma Atlet and RSCM Kiara Ultimate). Having intent (related to the risk perception, outcome expectancies, and self-efficacy) and having a plan can positively influence the behavior of people undergoing isolation, resulting in better compliance to health protocols. The understanding gained from this study can be used to improve strategies related to compliance with health protocols against COVID-19 in the communities.

## Introduction

The COVID-19 pandemic that has occurred over the last 2 years around the world presented great challenges not only to health workers, but also to the economy, government, education, and many other sectors in society (1, 2). Based on the latest data from WHO as of the time of writing (April 13, 2022), there have been 499,119,316 confirmed cases of COVID-19, with a total of 6,185,242 deaths. Indonesia has recorded 6,036,909 confirmed cases of COVID-19 and 155,746 deaths (3). The high transmission rate of COVID-19 continues to be a concern, especially the Delta and Omicron variant which was found to have a much higher transmission rate than the first strain encountered at the beginning of the pandemic. Due to its high transmission rate, various interventions to prevent the transmission of COVID-19 are also continuously being developed. These include physical distancing, self-isolation, quarantine, and health protocols. Vaccination program has also been implemented and is one of the main strategies currently used to control the COVID-19 pandemic. Although the vaccination program will run continuously until the transmission rate in the community decreases, prevention strategies in the form of health protocols, such as regularly washing hands, wearing masks, maintaining distance, avoiding crowds, as well as limiting mobility and interaction, still need to be carried out regularly. The CDC also recommends that vaccinated people should continue to comply with health protocols to prevent transmission (4, 5). Dewi and Probandari (6) also found a significant association between compliant behavior to health protocols, such as wearing masks and physical distancing outside the home and at the workplace, and the COVID-19 rapid test results.

Due to the importance of health behavior in the prevention of disease, various forms of health promotion continue to be conducted to achieve behavior change in terms of compliance with health protocols at the community level. However, community compliance level to the health protocols remains low in Indonesia. Based on a survey on community compliance with health protocols conducted by AC Nielsen with UNICEF, in six major cities in Indonesia, there were only 31.5% of respondents who performed all health protocol behaviors (including wearing masks, maintaining distance, and washing hands) in a disciplined manner. Others performed two of the three health protocol behaviors (36%), one of the three health protocol behaviors (23.2%), or did not comply with the health protocols at all (9.3%) (7). Fuady et al. (8), who conducted a study in Indonesian youths, also found that despite having good knowledge and attitude, in practice the results were significantly different. Fuady et al. (8) found that in Indonesian youth, the non-compliance rate to the health protocols was high, suggesting that knowledge and attitude alone are not enough to make a person perform health behaviors, particularly related to preventive strategies against COVID-19.

To achieve effective behavioral changes, it is necessary to identify the behavioral determinants that can be potentially modified and used as targets for intervention. Common obstacles that often keeps people from doing behavioral changes, let alone doing it consistently, are whether or not there is an intention to do the behavior and the gap between having intent and doing the behavior. A socio-cognitive approach, known as Health Action Process Approach (HAPA), can be used to understand the mechanism for someone to have intents and understand the gap between intent and behavior. HAPA helps to bridge and look for more specific determinant factors on how intent emerges into sustained behavioral change. HAPA distinguishes the two processes leading to health behavioral change, namely the pre-intentional motivation process and the post-intentional volitional process. In the motivational phase of HAPA, three socio-cognitive components influence the emergence of intent to change behaviors. The three components consist of expectations of the desired outcome, self-efficacy to make behavioral changes, and perception of personal risk. Expectations of the desired outcome may be in the form of social, physical, or emotional outcome expectancies. Self-efficacy is a person's belief in his capacity to perform the desired behavior. Risk perception is the identification and interpretation of a person's health risks, whether as specific diseases or non-specific conditions. In the volitional phase, two main components implicate the change after the intent emerges, which include planning and self-efficacy (both for maintaining behavior and recovery). Planning consists of two things: planning for actions, such as when, where, and how to act, and planning for coping that will be performed if there are obstacles encountered. Self-efficacy in the volitional phase includes a person's belief in one's capacity to maintain new behaviors through various coping mechanisms in dealing with the obstacles, as well as to reconduct the expected behavioral change if one fails. Moreover, there is action control that may also influence the behavioral changes, which is a self-regulatory strategy done when the behavior has already taken place and been continuously evaluated (9–15).

This study used HAPA to understand the complexity of compliance issues to health protocols. Previously, HAPA has also been used in several studies on health behaviors related to COVID-19, such as study conducted by Lao et al. (16) that found both motivation and volitional factors included in HAPA might improve compliance with several health protocols related to COVID-19, i.e., wearing facemask and handwash. Hamilton et al. (17) also used HAPA to assess social distancing behavior during the pandemic, and found that both processes from HAPA can be used to understand the behavior. Beeckman et al. (14) who also used HAPA as the framework for the study found the same results. Another study by Duan et al. (15) also identified some social-cognition determinants by integrating the theory of planned behavior, health knowledge, and HAPA on three health behaviors related to COVID-19. The study found that intents might be predicted by motivational self-efficacy, attitude and subjective norms, while behaviors might be predicted by health knowledge and action control, and also mediated by planning from volitional self-efficacy (15).

Although several studies have been found examining HAPA on health behaviors related to COVID-19, until this writing was made, no studies were found examining HAPA in the specific population, namely people who are undergoing isolation in health facilities, especially in Indonesia. In Indonesia, the government has provided some isolation facilities where people might undergo isolation and were guaranteed that they will receive masks, available handrub, be supervised continuously, and share appropriate rooms with some distance with other people. With the condition that all the supplies needed were available, this study tried to learn about the mechanism related to the compliance behavior in that specific population. Therefore, this study was conducted, aiming to identify the psychosocial determinants and examine the mediation mechanisms of the compliance with COVID-19 health protocols among people undergoing isolation in health facilities that specifically treat COVID-19 cases in Jakarta, Indonesia. The understanding are important to be known and may be used in developing future programs that targeted the compliance with health protocols of COVID-19 more specifically.

In this study, it was hypothesized that in people undergoing isolation in health facilities related to COVID-19 where the facilities needed where provided, there can be found direct association between self-efficacy in taking actions, outcome expectancies, and risk perception with intents. Moreover, this study also hypothesized that having intents has direct association with planning, planning has direct association with compliance to health protocols, and self-efficacy in maintaining behavior has a direct association with compliance and planning. Moreover, this study also hypothesized that planning mediate intention and compliance to health protocol.

## Materials and Methods

### Study Design

**Figure 1:**
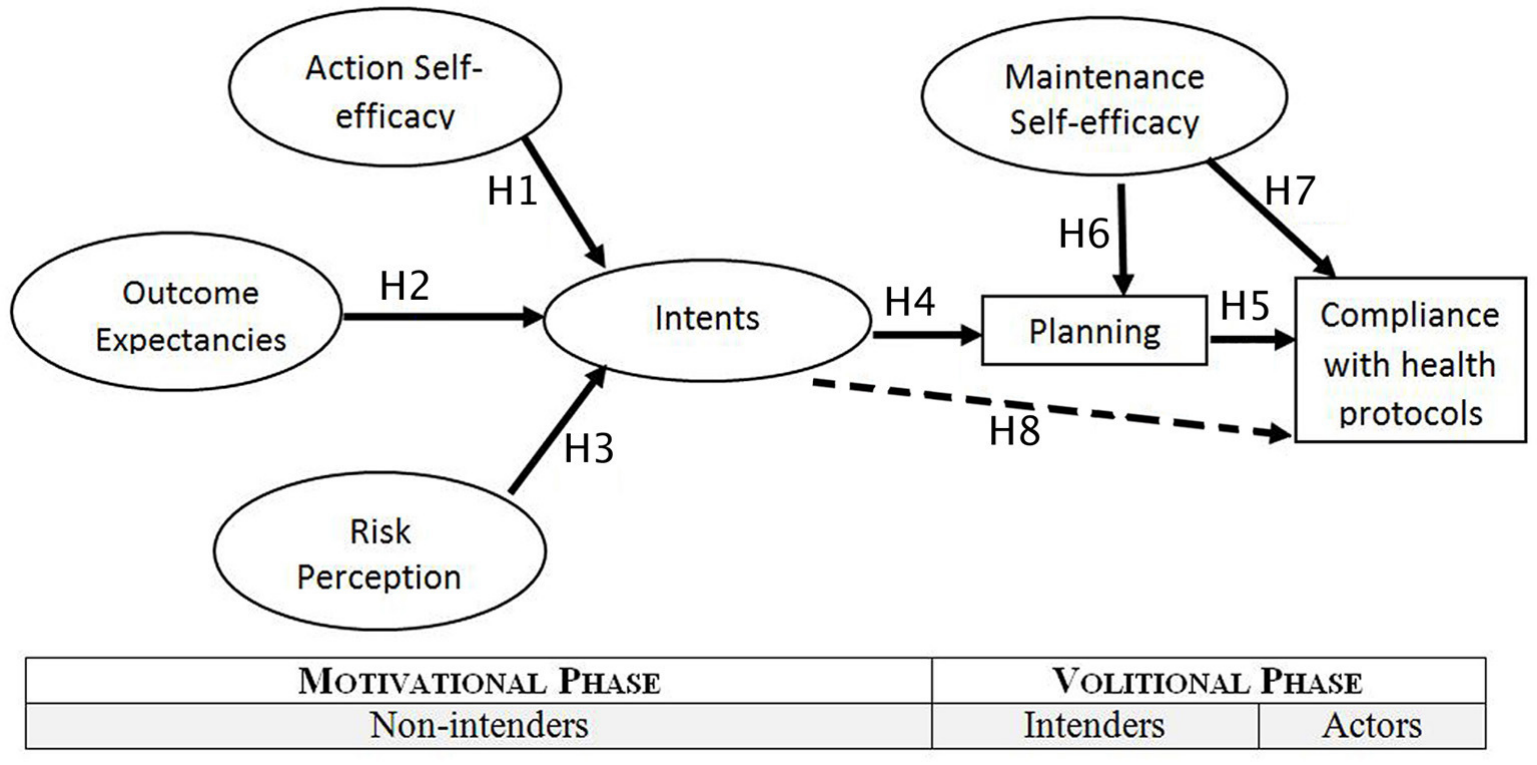
Hypothesis model in this study, adapted from Schwarzer (18), with permission.

This is a cross-sectional study, that used HAPA to understand the process leading to behavior change, i.e., compliance with health protocols. There were eight hypotheses tested in this study, as listed in Figure 1 below, including H1: Self-efficacy in taking actions has a direct association with intents; H2: outcome expectancies have a direct association with intents; H3: risk perception has a direct association with intents; H4: intents have a direct association with planning; H5: planning has a direct association with compliance; H6: Self-efficacy in maintaining behavior has a direct relationship with planning; H7: Self-efficacy in maintaining behavior has a direct association with compliance. Furthermore, this study also hypothesized that planning will mediate intention and compliance to health protocol, filling the intention-behavior gap in this community (H8).

### Participants

This study included individuals who had been confirmed of having COVID-19, and were undergoing isolation at the health facilities for COVID-19 in Jakarta, Indonesia (Wisma Atlet Kemayoran and Kiara Ultimate RSCM Jakarta) during the data collection period (October 19–26 2020). The inclusion criteria for this study was all people who were undergoing isolation at the health facilities for being confirmed of having COVID-19. The exclusion criteria was the subjects who were in a bad condition that affecting their understanding about the study and having difficulties filling out the distributed questionnaire. The *a-priori* sample size was determined based on the calculation formula for Structural Equation Modeling (SEM), with anticipated effect size 0.1, desired statistical power level 0.8, number of latent variables 7, number of observed variables 5, and probability level 0.05. Based on the calculation formula (19), the minimum sample size required to detect the effect is 1,808 samples, with a minimum of 805 samples for the model structure. In this study, subjects who met the inclusion and exclusion criteria were included until the time limit was over (the data collection period for this study was limited to 8 days). As many as 1,584 subjects completed the study, including 865 men and 719 women over the age of 18 years old.

### Procedures

The medical staffs worked in Wisma Atlet Kemayoran and Kiara Ultimate RSCM Jakarta were included to explain about the study to the respondents during the data collection period. The respondents who were selected based on the inclusion and exclusion criteria listed above were being informed about the aim of the study, steps to fill the questionnaire distributed, and the confidentiality issues. All of the participants who agree to join in this study have given the permission by signing in the online form of the informed consent form. The questionnaire in this study was distributed using an electronic form, following the regulations of the health facility where the study was conducted, to avoid COVID-19 transmission. When completing the questionnaire, respondents were accompanied by the research team.

### Research Instrument

To test the hypotheses, a survey questionnaire adapted from the questionnaire made by Schwarzer (11) was used in this study. The questionnaire was developed by a team of experts from the Faculty of Medicine, Universitas Indonesia—Dr. Cipto Mangunkusumo General Hospital, and survivors. The development was conducted by first creating, distributing, and collecting data using a pre-test questionnaire, followed by modifications to develop a formal questionnaire which was then distributed for study data collection. The developed questionnaire contains several questions on how often respondents follow quarantine guidelines and is scored using a 5-point Likert scale. Score is calculated based on the respondents' answers, with a score of 1 given if the respondent answered 'never' up to a score of 5 if the respondent answered 'always' on the statement item for the behavior. The distributed questionnaire consists of three parts, covering the characteristics of the interviewees (gender, age, and level of education), measurements of risk perception, outcome expectancies, self-efficacy, intent, planning, and compliance, as well as scores given by respondents (Appendix A). The questionnaire distributed may need ~15–20 min to be fulfilled.

### Statistical Analysis

This study used the SEM multivariate data analysis technique. There are two types of variables in structural equation modeling: latent and measured variables. Latent variables are variables that cannot be observed or measured directly. In this study, the latent variables that were assessed included risk perception, outcome expectancies, self-efficacy, intent, planning, and compliance. Measured variables, also known as indicators, are variables that can be observed or measured directly (20, 21).

The structural equation modeling consists of measurement/outer model and structural/inner model. The measurement model is used to explain the association between measured variables and latent variables, while the structural model describes the association between latent variables. In the structural/inner model, there are exogenous latent variables that can predict other variables and endogenous latent variables that are predicted by other variables and show their effects. An approach that can be used for SEM is Partial Least Squares (PLS) which is a path modeling approach without any assumptions about the data distribution. PLS-SEM has several advantages such as being suitable when the sample size is limited, when the data distribution is skewed/asymmetrical, as well as when the prediction accuracy is desired. The PLS-SEM approach can be performed using the SmartPLS application software (20–23). This study also assessed the relationship between demographic factors of the participants (education and sex variables) as control variables and the compliance behaviors.

### Measurement/Outer Model

The analysis of the outer model is important because the validity and reliability of the association in the inner model are also determined by the outer model. The outer model analysis assesses internal consistency reliability, indicator/measured variable reliability, convergent validity, average variance extracted (AVE), and discriminant validity. Internal consistency reliability analysis based on the association between the variables observed in this study was performed using Cronbach's alpha and composite reliability measurements. A value closer to one indicates better reliability. In this study, Cronbach's alpha and composite reliability values of >0.7 are acceptable. Indicators with outer loading values >0.7 were accepted, while those <0.4 were omitted (24, 25).

Loading factor indicator, composite reliability, and AVE were observed to assess convergent validity. An AVE value >0.5 is considered adequate for convergent validity, and can also be used to assess discriminant validity. Assessment of discriminant validity was performed to ensure that the latent constructs used in this study are truly unrelated and do not measure the similar construct as other variables (24–26). In this study, discriminant validity was calculated using the Fornell and Larcker criterion and the Heterotrait-monotrait (HTMT) criterion from Henseler (26, 27). According to the Fornell-Larcker criterion, the latent variables should be better at explaining the variance of their indicator variable than other latent variables and are indicated by a larger square root of the AVE value. According to the HTMT criterion, a value closer to 1 indicates a lack of discriminant validity (25, 26).

### Structural/Inner Model

Analysis of the inner model quality includes an assessment of the coefficient of determination (*R*^2^), path coefficient, and effect size (*f*^2^). R-square (*R*^2^) or the coefficient of determination is used to assess the predictive power of the model proposed in the hypothesis and see the combined effect of the exogenous variables on endogenous variables. The *R*^2^ values range from 0 to 1, with a value closer to one indicating a better model (24).

Analysis of the path coefficient can describe the association between variables in the hypothesis. The path coefficient values range from −1 to +1, with a coefficient closer to 1 indicating a stronger association, whether the association is positive or negative. The significance can then be obtained through standard error using bootstrapping technique. *P*-Value of <0.05 indicates a significant prediction between independent and dependent variables. The effect size (Cohen's *f*^2^) was determined by assessing the change in the coefficient of determination when a specific variable in the model was omitted, as well as by estimating two PLS path models (the complete model that fits the hypothesis and model with some exogenous variables that have been omitted). The effect size of each association was determined as follows: 0.02 (small effect), 0.15 (medium effect), and 0.35 (large effect) (24).

### Goodness of Fit

The Goodness of Fit of the models can be measured by using SmartPLS (23). The measurements include Standardized Root Mean Square Residual (SRMR), Normal Fit Index (NFI), and Root Mean Square Theta (RMS_theta). SRMR shows how big the difference of root mean square between observed and expected correlations is. The recommended SRMR value is <0.10 or 0.08 in the conservative version. In this study, the SRMR value limit used was 0.08. The RMS_theta values (root mean square residual covariance) were also assessed with a value <0.12 indicating a well-fitting model, and a higher value indicating a lack of fit in the model. The expected NFI value is ≤ 1, with a value closer to 1 indicating a very good fit, and a value <0.9 usually representing an acceptable fit (28, 29).

### Mediation Analysis

Mediation analysis was made to know the process or “how” the relation between the two variables, to better understand the mechanism the effect happens. In this study, mediation analysis were made with smartPLS with the variables: intention, compliance and planning (23, 30). The total effect, direct and indirect effect will be presented in the table with the coefficient and significance value.

### Ethical Approval Statement

This study has obtained ethical clearance from the Faculty of Medicine, Universitas Indonesia on April 27, 2020, with reference number KET-444/UN2.F1/ETIK/PPM.00.02/2020.

## Results

### Demographic Characteristics of Participants

Table 1 shows the demographic characteristics of the participants in this study. The participants consisted of 55% males and 45% females with the majority (61%) having completed secondary high school. Most of the participants were in the range of 18–35 years old (65.49%). The marital status of the participants were 53% married, 42% unmarried, and 5% divorced. For control variables, there were no significant relationship found to the compliance behaviors (*P* = 0.365 for education; *P* = 0.263 for sex variables).

**Table 1:** Demographic characteristics and difference in perceived compliance to health protocol among subjects (*n* = 1,584).

**Group**	**Descriptive**		**Compliance to health protocol**	
	**Frequency**	**%**	**Mean**	**SD**
**Age (years old)**				
18–35	1,038	65.49	4.333	0.045
36–55	492	31.17	4.235	0.071
>55	54	3.68	4.122	0.223
**Sex variables**				
Man	865	55%	4.193	0.057
Woman	719	45%	4.262	0.051
**Education**				
Junior high school	150	9%	4.219	0.113
High school	970	61%	4.218	0.058
Bachelor's degree	405	26%	4.261	0.071
Master's/Doctoral degree	59	4%	3.791	0.215
**Marital status**				
Unmarried	666	42%	4.161	0.049
Married	847	53%	4.274	0.066
Divorced or widow/widower	71	5%	4.272	0.155

### Research Variables

Table 2 describes the variables and the measurement indicators used in this study. There was no missing data during the data collection phase, with each variable having a minimum value of 1 and a maximum value of 5.

**Table 2:** Research variables and measurement indicators.

**Variables**	**Missing**	**Min**	**Max**	**Mean**	**SD**
Risk1	0	1	5	1.871	1.309
Risk2	0	1	5	2.153	1.401
Risk3	0	1	5	1.921	1.331
Risk4	0	1	5	2.174	1.449
Risk5	0	1	5	1.437	0.895
Expectancy1	0	1	5	4.643	0.931
Expectancy2	0	1	5	4.622	0.975
Expectancy3	0	1	5	4.621	0.952
Expectancy4	0	1	5	4.588	0.961
Expectancy5	0	1	5	4.542	0.981
EfficacyAction1	0	1	5	4.657	0.841
EfficacyAction2	0	1	5	4.559	0.919
EfficacyAction3	0	1	5	4.580	0.912
EfficacyAction4	0	1	5	4.612	0.893
EfficacyAction5	0	1	5	4.598	0.887
EfficacyAction6	0	1	5	4.448	0.981
EfficacyAction7	0	1	5	4.333	1.058
Intent1	0	1	5	4.722	0.757
Intent2	0	1	5	4.740	0.793
Intent3	0	1	5	4.658	0.846
Intent4	0	1	5	4.568	0.905
Intent5	0	1	5	4.679	0.835
Plan1	0	1	5	4.380	1.031
Plan2	0	1	5	4.388	1.043
Plan3	0	1	5	4.522	0.941
Plan4	0	1	5	4.613	0.899
Plan5	0	1	5	4.568	0.938
Plan6	0	1	5	4.398	1.035
Plan7	0	1	5	4.493	0.966
Plan8	0	1	5	3.965	1.245
EfficacyMaintn1	0	1	5	4.114	1.183
EfficacyMaintn2	0	1	5	4.088	1.169
EfficacyMaintn3	0	1	5	4.280	1.099
EfficacyMaintn4	0	1	5	4.526	0.958
EfficacyMaintn5	0	1	5	3.958	1.294
Adherence1	0	1	5	3.600	1.376
Adherence2	0	1	5	4.410	1.012
Adherence3	0	1	5	4.485	0.968
Adherence4	0	1	5	4.720	0.768
Adherence5	0	1	5	4.297	1.300
Adherence6	0	1	5	4.266	1.064
Adherence7	0	1	5	4.549	0.898
Adherence8	0	1	5	4.037	1.209

### Measurement Model

#### Internal Consistency Reliability

Table 3 below shows the Cronbach's alpha and composite reliability value of the variables. For compliance, intent, outcome expectancies, planning, perception of risk, and self-efficacy in action and maintaining behaviors, all of the Cronbach's alpha and composite reliability values are above the expected value (0.7), indicating good internal consistency.

**Table 3:** Reliability and convergent validity of the whole measurement model.

	**Cronbach's alpha**	**Rho_A**	**Composite reliability**	**AVE**
Compliance behavior	0.877	0.879	0.907	0.621
Intention	0.932	0.932	0.949	0.787
Outcome expectancy	0.939	0.940	0.954	0.804
Risk perception	0.808	0.818	0.864	0.561
Self-efficacy for action	0.934	0.936	0.947	0.718
Self-efficacy for maintenance	0.869	0.877	0.906	0.658
Planning	0.941	0.943	0.951	0.711

#### Reliability Indicator

Table 4 shows the results of the indicator reliability of the measurement model as a whole with outer loading values of more than 0.7. Initial analysis found outer loading values that were below 0.7, thus did not fulfill the expected limits, and some variables were removed from the construction model. The variables removed include “adherence1” and “adherence5.”

**Table 4:** Indicator reliability/outer loading.

	**Compliance behavior**	**Intention**	**Outcome expectancy**	**Risk perception**	**Self-efficacy for action**	**Self-efficacy for maintenance**	**Planning**
Adherence2	0.745						
Adherence3	0.801						
Adherence4	0.802						
Adherence6	0.817						
Adherence7	0.832						
Adherence8	0.726						
EfficacyAction1					0.829		
EfficacyAction2					0.818		
EfficacyAction3					0.871		
EfficacyAction4					0.866		
EfficacyAction5					0.879		
EfficacyAction6					0.853		
EfficacyAction7					0.811		
EfficacyMain1						0.785	
EfficacyMain2						0.818	
EfficacyMain3						0.872	
EfficacyMain4						0.845	
EfficacyMain5						0.728	
Expectancy1			0.877				
Expectancy2			0.890				
Expectancy3			0.903				
Expectancy4			0.916				
Expectancy5			0.897				
Intent1		0.870					
Intent2		0.903					
Intent3		0.910					
Intent4		0.861					
Intent5		0.891					
Plan1							0.818
Plan2							0.838
Plan3							0.859
Plan4							0.862
Plan5							0.871
Plan6							0.880
Plan7							0.878
Plan8							0.729
Risk1				0.766			
Risk2				0.754			
Risk3				0.731			
Risk4				0.757			
Risk5				0.735			

#### AVE and Convergent Validity

The AVE value was between 0.621 and 0.804, which is above the expected value (0.5). The composite reliability and AVE values show sufficient convergent validity in the measurement model created.

#### Discriminant Validity

Table 5 below describes the correlation between latent variables by comparing the square root of each AVE with the correlation coefficient of other latent variables. The square root of each variable's AVE in this study was larger than the correlation with other latent variables, thus the discriminant validity is accepted, based on the Fornell Larcker Criterion. The HTMT criterion was also used to calculate discriminant validity. In Table 6 revealed an issue with collinearity between the latent variables intent, and self-efficacy for action, indicating that there is overlap between the two latent variables. There was no overlap between other items.

**Table 5:** Discriminant validity- Fornell-Larcker criterion.

	**Compliance behavior**	**Intention**	**Outcome expectancy**	**Risk perception**	**Self-efficacy for action**	**Self-efficacy for maintenance**	**Planning**
Compliance behavior	***0.788***						
Intention	0.713	***0.887***					
Outcome expectancy	0.535	0.686	***0.897***				
Risk perception	−0.080	−0.082	−0.126	***0.749***			
Self-efficacy for action	0.777	0.856	0.633	−0.106	***0.847***		
Self-efficacy for maintenance	0.683	0.631	0.510	−0.076	0.720	***0.811***	
Planning	0.744	0.704	0.503	−0.076	0.771	0.672	***0.843***

*Bold and italics, the square root of AVE*.

**Table 6:** Discriminant validity—Heterotrait-monotrait ratio (HTMT).

	**Compliance behavior**	**Intention**	**Outcome expectancy**	**Risk perception**	**Self-efficacy for action**	**Self-efficacy for maintenance**	**Planning**
**Compliance behavior**							
Intention	0.786						
Outcome expectancy	0.588	0.734					
Risk perception	0.094	0.091	0.143				
Self-efficacy action	0.858	**0.915**	0.674	0.120			
Self-efficacy maintenance	0.779	0.694	0.559	0.095	0.794		
Planning	0.819	0.749	0.533	0.090	0.822	0.739	

*Collinearity issue between the latent variables intent and self-efficacy for action, indicating that there is overlap between the two latent variables*.

### Structural Model

#### Determination Coefficient

Table 7 shows the determination coefficient or strength of the predictive model created for behavior, intent, outcome expectancies, planning, and risk perception. The model could explain the variations in 61.5% of compliance, 76.8% of intent, and 58.1% of planning.

**Table 7:** Determination coefficient (*R*^2^).

	**R square**	**R square adjusted**
Compliance behavior	0.615	0.614
Intention	0.768	0.768
Planning	0.581	0.614

#### Path Coefficient

Table 8 shows the path coefficient for all paths proposed in the study model. All seven path coefficients proposed in this study were significant. The results in Table 8 show that and self-efficacy for action, outcome expectancies, and risk perception is related to intent, supporting H1 (β = 0.705; *P* = 0.000; *T*-value = 19.602), H2 (β = 0.243; *P* = 0.000; *T*-value = 6.467), and H3 (β = 0.023; *P* = 0.048; *T*-value = 1.981). Furthermore, intent has a significant effect on planning, and planning has a significant effect on compliance to with health protocols, supporting H4 (β = 0.465; *P* = 0.000; *T*-value = 11.533) and H5 (β = 0.519; *P* = 0.000; *T*-value = 11.435). Additionally, self-efficacy in maintaining behaviors was also found to have a positive effect on planning and compliance to health protocols which supports H6 (β = 0.378; *P* = 0.000; *T*-value = 10.062) and H7 (β = 0.334; *P* = 0.000; *T*-value = 7.590). Compared to results by Hamilton et al. (17), this study found the same results in how self-efficacy may predict intention and having intents predicted behavior [in Hamilton et al., (17) the results were β = 0.314, *P* < 0.001 and β = 0.261, *P* = 0.026, respectively]. However, Hamilton et al. (17) did not found that risk perception may predict intention significantly (β = 0.150, *P* = 0.077) (17).

**Table 8:** Result of final model hypothesis.

	**Beta coefficients**	**Standard deviation**	***T*** **statistics**	***P*** **-value**
H1: Self-efficacy for action → Intention	0.705	0.036	19.602	0.000
H2: Outcome expectancy → Intention	0.243	0.038	6.467	0.000
H3: Risk Perception → Intention	0.023	0.012	1.981	0.048
H4: Intention → planning	0.465	0.040	11.533	0.000
H5: Planning → compliance to protocol	0.519	0.045	11.435	0.000
H6: Self-efficacy for maintenance → Planning	0.378	0.038	10.062	0.000
H7: Self-efficacy for maintenance → Compliance to protocol	0.334	0.044	7.590	0.000

**Figure 2:**
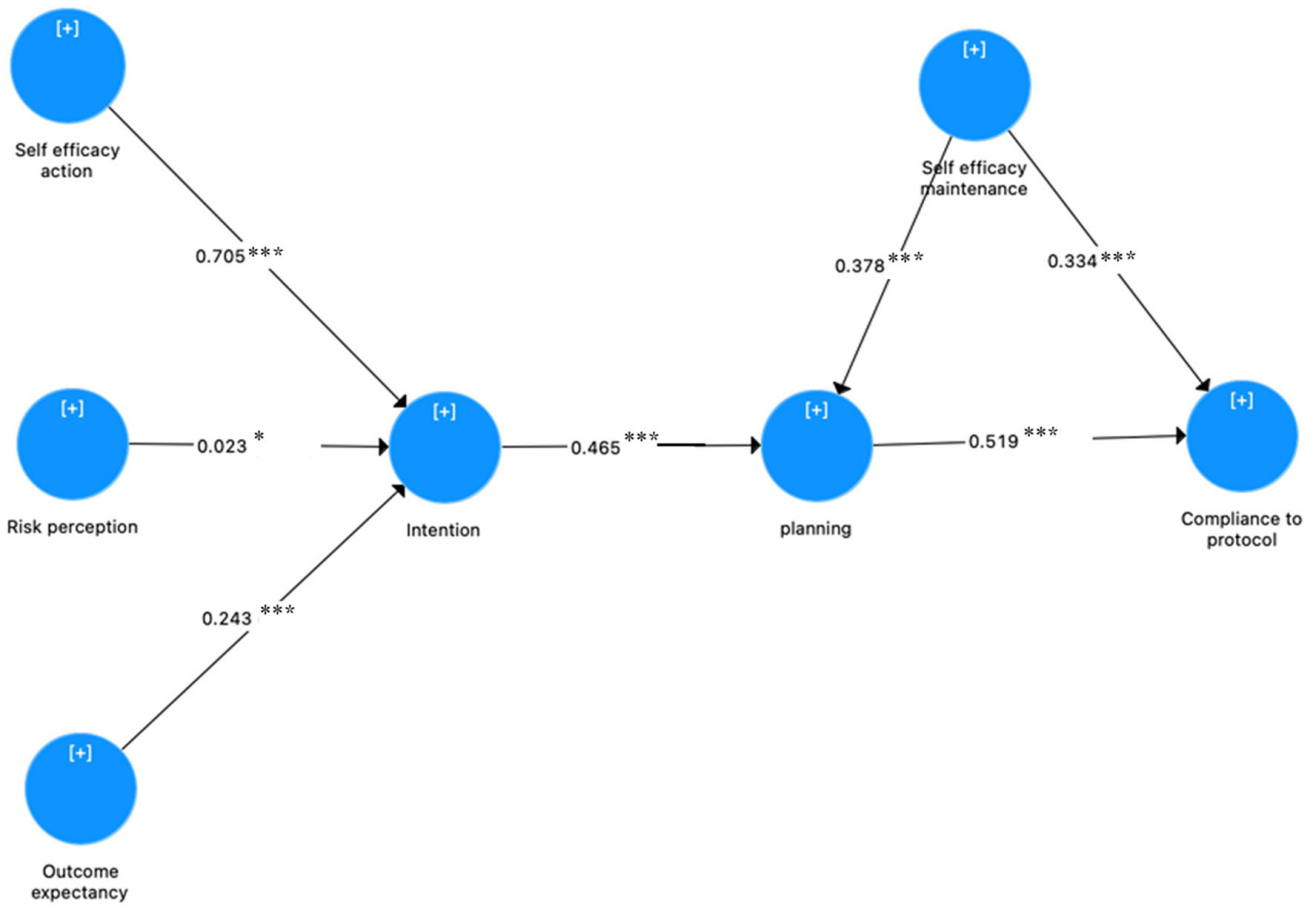
Final model path coefficient (*indicating *P* < 0.05; ***indicating *P* < 0.001).

The final path coefficient from compliant behaviors to health protocols is described in Figure 2. The figure was obtained from SmartPLS software (23). Table 9 describes the direct, indirect and total effects of the variables in the HAPA model. The total effect of a latent variable on the HAPA model is the combination of direct and indirect effects. The results showed that self-efficacy action had the greatest direct effect on intention (β_*total*_ = 0.705, *P* < 0.000) and self-efficacy maintenance had the greatest total effect on compliance (β_*total*_ = 0.531, *P* < 0.000). Higher level of intention and self-efficacy maintenance gave rise to the higher level of planning, and planning also had a high effect on compliance (β_*total*_ = 0.519, *P* < 0.000). Self-efficacy action and intention had a moderate total effect on compliance (β_*total*_ = 0.170, *P* < 0.000 and β_*total*_ = 0.241, *P* < 0.000). Outcome expectancy had a small total effect on compliance (β_*total*_ = 0.059, *P* < 0.000).

**Table 9:** The direct, indirect and total effects of the variables in the HAPA model.

**Latent variables**	**Direct effects**	**Indirect effects**							**Total effects**						
		**SEA**	**RP**	**OE**	**IT**	**PL**	**SEM**	**CL**	**SEA**	**RP**	**OE**	**IT**	**PL**	**SEM**	**CL**
Self-efficacy action	0.705^***^	–	–	–	–	0.327^***^	–	0.170^***^	–	–	–	0.705^***^	0.327^***^	–	0.170^***^
Risk perception	0.023^*^	–	–	–	–	0.011	–	0.006		–	–	0.023^**^	0.011	–	0.006
Outcome expectancies	0.243^***^	–	–	–	–	0.107^***^	–	0.113^***^	–	–	–	0.243^***^	0.113^***^	–	0.059^***^
Intention	0.465^***^	–	–	–	–	–	–	0.241^***^	–	–	–	–	0.465^***^	–	0.241^***^
Planning	0.519^***^	–	–	–	–	–	–	–	–	–	–	–	–	–	0.519^***^
Self-efficacy maintenance	0.334^***^							0.196^***^	–	–	–	–	0.378^***^	–	0.531^***^

*SEA, self-efficacy action; RP, risk perception; OE, outcome expectancy; IT, intention; PL, planning; SEM, self-efficacy maintenance; CL, compliance.*

* *Indicates P < 0.05.*

** *Indicates P < 0.01.*

*** *Indicates P < 0.001.*

#### Effect Size

Table 10 shows the effect sizes of H1-H7. A medium effect size was found for H2 (outcome expectancies to intents), H4 (intents to planning), H6 (self-efficacy in maintaining behavior to planning), and H7 (self-efficacy in maintaining behavior to compliance). A large effect size was found for H1 (from self-efficacy for action to intent) and H5 (planning to compliance).

**Table 10:** *F*-square.

	**Compliance to protocol**	**Intention**	**Planning**
Intention			0.310
Outcome expectancy		0.152	
Risk perception		**0.002**	
Self-efficacy for action		1.283	
Self-efficacy for maintenance	0.159		0.206
Planning	0.384		

*Risk perception has a F2 effect size of 0.002, which is smaller than the Cohen's proposed lower limit of 0.02 (24). The low F2 effect size, combined with the near non-significance of the intention effect at Table 8 indicated that risk perception and intention might have a linear relationship in our dataset*.

#### Goodness of Fit

Table 11 shows the results of the model fit measurement, including the saturated model and estimated model. In this study, the SRMR value was 0.045 which is below the expected value of 0.08. Therefore, it can be concluded that the model fulfills the criteria for a good model fit. The RMS_theta value also had a value below 0.109, indicating a well-fitting model. The NFI value was also close to 1, which indicates an accepted fit (28).

**Table 11:** Goodness of fit—structural/inner model.

	**Saturated model**	**Estimated model**
SRMR	0.045	0.060
d_ULS	1.713	3.097
d_G	0.661	0.722
Chi-square	6615.444	6985.731
NFI	0.878	0.871
RMS theta	0.109	

### Mediation Analysis

**Figure 3:**
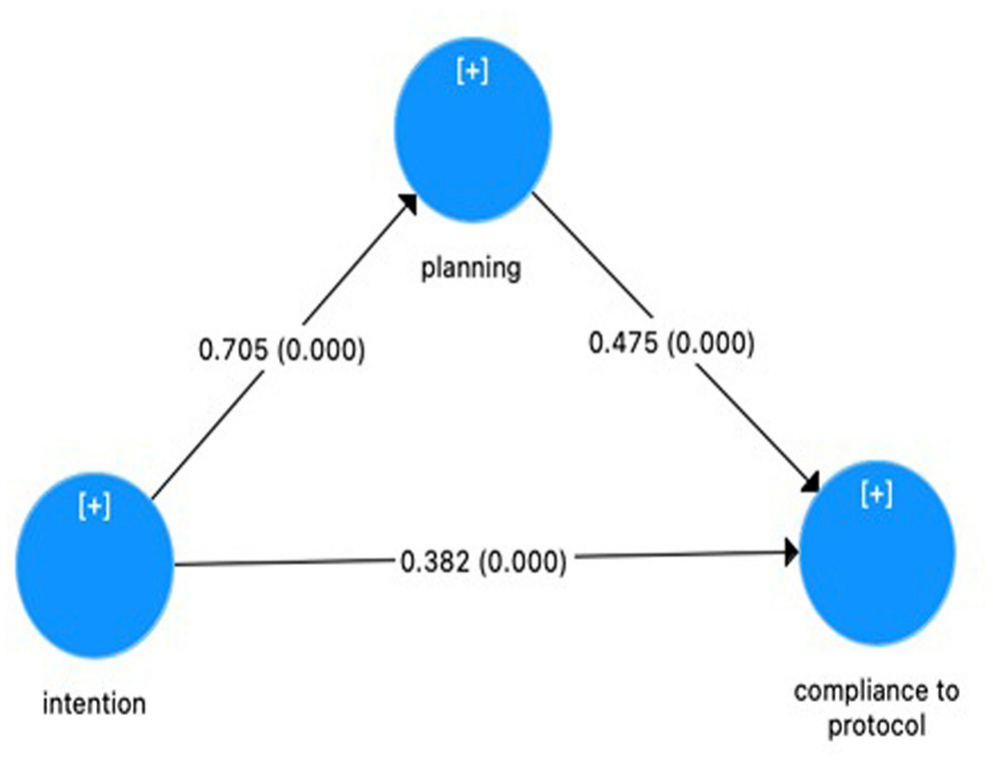
Mediation analysis.

The total effect which is the effect of having intents on compliance without the involvement of planning was significant (β = 0.716; *P* = 0.000). Moreover, further analysis also found that having intents also have a significant impact on compliance in the presence of planning as the mediator (β = 0.382; *P* = 0.000) and significant impact of having intents on compliance through planning (β = 0.334; *P* = 0.000). These results can be seen in Table 12. Figure 3 shows that planning partially mediates an effect from intent to be compliant toward health protocols significantly.

**Table 12:** Mediation analysis.

**Total effect (intention to compliance)**		**Direct effect (intention to compliance)**		**Indirect effects of intention on compliance**				
**Coefficient**	***P*** **-value**	**Coefficient**	***P*** **-value**		**Coefficient**	**SD**	***T*** **-value**	***P*** **-value**
0.716	0.000	0.382	0.000	H8: I -> P -> C	0.334	0.039	8.506	0.000

## Discussion

This study was conducted to fill the gap by trying to look more specifically about the behavioral change, i.e., compliance to health protocols, in people who are undergoing isolation in the health facilities for COVID-19 in Indonesia. The participants in this study include confirmed COVID-19 cases. Indrayathi et al. (31) found that COVID-19 test histories, either with PCR or rapid antibody test, and also knowing someone who had been confirmed positive or died from COVID-19 were related significantly with adherence to prevention measures. In people undergoing isolation which mean that they have been confirmed of having COVID-19, it may be suspected that they will be more comply to the health protocols. They will see their surroundings who are using full health protocols in the health facilities, in which human behavior will be influenced by the culture, including how they perceive other people will think about them, and also how they see people around them behaving (32). So, this condition in the health facilities may influence them to be more motivated and planning to comply to the behaviors. However, it was still unclear because they may also feel as they have experienced COVID-19, they will be more free and feel careless to comply. On the other hand, the health facilities where the isolation takes place were also providing the facilities needed, such as continuous observation, handrub, and they will be more exposed to information/education related to COVID-19. The behaviors assessed in the study including not going out to do activities outside the quarantine area, and it is a must in healthcare facilities. They will also separate themself or keep some distance from other people as the bed were organized to be in some safe distance. They will also have to wear a mask all the time when there are other people in the room, and the behaviors will be confirmed and reminded when the medical staffs visit them in the daily round. In the health facilities, they will not also share personal tools with others as they may usually do in their daily life outside the isolation place and they will also be assessed daily for the body temperature and symptoms. This study was conducted to look more closely on the behavior changes in these conditions, aiming to provide information about the mediating mechanisms and determinants of compliance with health protocols in people who have been provided by the facilities needed.

This study found that the model proposed is a good fit, and may explain the determinants for behavioral changes among people undergoing isolation in COVID-19 healthcare facilities (Wisma Atlet and RSCM Kiara Ultimate), starting from one's risk perception to COVID-19 transmission, their expectation of the outcome, and their confidence of their own capability to comply, thus allowing them to form intent, plan, and start acting to comply to health protocols and to continuously maintain such compliance. Luszcynska et al. (33) who also used HAPA as the framework to assess compliance to handwashing behavior, found that risk perception and outcome expectancies were linked but only indirectly to the expected behavior. That study found that self-efficacy and self-monitoring or action control are more consistent in predicting expected health behavior (33). Unfortunately, in this study, the action control were not assessed. However, the results for the risk perception and outcome expectancies were also found to be the same in this study, and the self-efficacy's result is also consistent. Based on the results of this study, both forms of self-efficacy (to perform and maintain behaviors) have a permanent effect on health behaviors and play an important role. In those with strong self-efficacy regarding their ability to perform the behavioral change, they also tend to have bigger intent toward compliance. This study also found that stronger intent will trigger stronger planning.

This result is also in-line with the statement from Bandura (34) regarding cognitive social theory in the effort to promote health and prevent diseases. Bandura (34) stated that among all determinants, confidence in self-efficacy plays an important role in personal change because it is needed to overcome obstacles faced in changing behaviors and becomes the foundation for self-motivation and action. This study is also in-line with a study of Isa et al. (35) that found inverse relation between self-efficacy scores to intent-behavior gap. They found in children with intention-behavior gap, they tend to have lower self-efficacy (35). Beeckman et al. (14) also found that self-efficacy were related to adherence to physical distancing as the behavior measured in the study, along with outcome expectancies, having intents and planning. However, in this study, the relation between outcome expectancies to compliance were not found to be large enough. Luc PT (36) which found no direct relation between outcome expectancies to intention in social entrepreneurial, suggested that outcome expectancies are flexible, related to others' support and recognition of the opportunities.

At the intender phase, an individual is already planning to change behaviors. Differ to study by Lao et al. (16) and Hamilton et al. (17) which found that planning did not mediate having intents to behavior change, this study found that planning mediate the two processes and may be used to understand the intention-behavior gap that often found when someone want to do behavior changes. After the behavior has been started, maintaining the behavior is almost as challenging as beginning to do the behavior (37, 38). It is unclear whether the changes in behavior can be maintained in the long term. However, self-efficacy in maintaining behavior are related to the compliance and is related to various factors, including personal factors such as age and level of education, as well as environmental factors such as obstacles and social support. To maintain self-efficacy for the long term, modifiable factors such as continuous social support are needed (39–41).

From the behavioral model proposed in this study, potential improvements can be identified in several points that have high benefits for change, thus the community can become more compliant toward health protocols to prevent COVID-19 transmission. With this knowledge, interventions can be implemented through various strategies targeted at increasing self-efficacy. Individuals who are doubtful toward self-efficacy can be given support and input through consultation. Those with low self-efficacy can be given a structured program to develop a strong sense of confidence in implementing or maintaining behaviors. Additionally, other strategies such as education, reflection on previous experiences, provision of behavior models, or interventions through mental imagery can also be performed. Programs to improve self-management abilities may also be beneficial, such as creating target behaviors that need to be achieved, assessing the situation when the behavior has been performed, and obtaining feedback or appreciation for each behavior that is following the target. The intervention strategies can vary between individuals, depending on each individual's preparedness toward change (10, 34, 42).

### Limitations

This study has some limitations. First, this study was limited to subjects undergoing isolation in healthcare facilities. Second, the instrument used in this study was self-reported, and it may affect the results because of the social desirability bias. The participants may also report their condition during their isolation (after being exposed to COVID-19) or report about their behavior usually before being exposed to COVID-19 and underwent the isolation in the facilities where the study was taken place. Third, the cross-sectional data used in this study also reduce the power of the study in forming a conclusion, as it may not give temporal relationship between the factors being hypothesized. With the cross-sectional study design, some recall bias may also be considered as limitation. Fourth, this study also did not portray the subject's past lifestyle or previous exposure to COVID-19 infection and/or other infectious diseases. Therefore, future studies may gather data longitudinally to investigate the effect of change and reciprocals between the construct models, comparing between individuals undergoing isolation in healthcare facilities and at home, and perform experimental studies that target changes in the HAPA construct individually. Future studies may also include more psychosocial determinants, such as psychosocial wellbeing, lifestyle, or social support, that were found related to difficulties in adherence behavior in a study conducted by Beeckman et al. (14). Moreover, although action control, which can be found in the original HAPA model suggested by Schwarzer et al. (12), was something crucial, but it was not included in the hypothesis or the path model in this study. This study also simplified the coping and action planning as “planning,” which includes both action and coping planning (13, 18). More specific HAPA construct which include action control and specify planning into action and coping planning may also be done in the future research.

## Conclusion

This study was conducted to fill the gap by trying to look more specifically about the behavioral change in the population who were undergoing isolation in health facilities related to COVID-19, especially in Indonesia. It can be concluded that intent, which related to the perception of risk, expected outcome, and self-efficacy has a positive influence on people undergoing isolation in healthcare facilities regarding their compliance with health protocols. Planning was also found mediates intention and compliance with health protocols. The understanding gained from this study can be used to improve strategies related to compliance with health protocols against COVID-19 in the communities, such as providing education, support, and consultation when needed.

## Data Availability Statement

The raw data supporting the conclusions of this article will be made available by the authors, without undue reservation.

## Ethics Statement

This study obtained ethical clearance from the Faculty of Medicine, Universitas Indonesia on 27th April 2020 under ethical clearance number KET-444/UN2.F1/ETIK/PPM.00.02/2020. The patients/participants provided their written informed consent to participate in this study.

## Author Contributions

KM, NA, AK, and PL conceptualized and designed. KM supervised the study and analyzed the data. KM, RK, LM, and LD collected the data. KM and AL wrote the manuscript and review. All authors have read and approved of the publication manuscript.

## Funding

This study was funded by Covid-19 research and innovation consortium from National Research and Innovation Agency Ministry of Research and Technology, Republic of Indonesia, with grant numbers 110/FI/PKS-KCOVID-19.F/VI/2020.

## Conflict of Interest

The authors declare that the research was conducted in the absence of any commercial or financial relationships that could be construed as a potential conflict of interest.

## Publisher's Note

All claims expressed in this article are solely those of the authors and do not necessarily represent those of their affiliated organizations, or those of the publisher, the editors and the reviewers. Any product that may be evaluated in this article, or claim that may be made by its manufacturer, is not guaranteed or endorsed by the publisher.

## Appendix

**Appendix A:** Overview of variables and psychometric data.

**Variable**	**Mean score (*n* = 1,584)**
**Risk perception**	
I think the rules during quarantine are too much, it's enough to just wear a mask	1.871
I think the reason for imposing quarantine on myself is not clear, there are still many people out there who are more deserving of quarantine	2.153
I went into quarantine because of social pressure, I was asked by people to do it while I didn't feel the need to do it myself	1.921
If asked to choose between doing quarantine or making a living, then I choose to make a living	2.174
I tend to disobey quarantine rules because they are too many and complicated	1.437
**Outcome expectancies**	
By doing quarantine I help the government reduce the number of COVID-19 infections in Indonesia	4.643
If I keep my distance or separate myself from family members, I am protecting my family from contracting COVID-19	4.622
By doing quarantine, I can rest and recover my health	4.621
By doing quarantine, I feel calm because it prevents other people from close contact and meeting	4.588
If I routinely monitor daily symptoms during quarantine, I can monitor my condition and seek medical help at the right time	4.542
**Intention**	
I am willing to obey the quarantine rules	4.722
I intend to always cover my nose and mouth when I cough or sneeze	4.740
I intend to use and clean my own personal tools such as cutlery, toiletries, bed sheets	4.658
I intend to always clean the surfaces of objects I touch such as cell phones, desks, door handles	4.568
I intend to always wash my hands with soap and running water or hand washing liquid after touching the face or object surface	4.679
**Action Self-efficacy**	
I'm sure I can do quarantine according to the required time	4.657
I'm sure I can undergo quarantine even though there are important obligations and responsibilities at this time	4.559
I feel sure I can wear a mask even if it's uncomfortable	4.580
I truly believe that I am capable of not sharing the usage of personal tools such as cutlery, towels and sheets with other people even though it is more difficult to do so	4.612
I seriously say I can always wash my hands with soap and running water or hand sanitizer, even if my hands become dry or sore	4.598
I'm sure that I can clean the surface of things that I often touch, such as tables, cell phones, doors	4.448
I certainly believe that I can monitor daily temperature, and cough and cold symptoms even though I have to fill the form	4.333
**Planning**	
I make a positive daily activity plan that can be done in quarantine	4.380
I make arrangements for the quarantine to be able to keep my distance or separate myself from other people	4.388
I plan to provide a mask and always wear a mask when there are other people in the room	4.522
I have a plan so that I don't forget to cover my mouth and nose when I cough or sneeze	4.613
I am planning a way to separate personal items such as cutlery, towels and bed linens so that they are separated from other people's belongings	4.568
I plan ways and schedules to clean the surfaces of objects that I touch frequently, such as tables, cell phones, doors	4.398
I have a plan on how to provide handwashing equipment and when to do it	4.493
I schedule to monitor the body temperature and symptoms daily, at 7 a.m. and 7 p.m.	3.965
**Maintenance Self-efficacy**	
I'm sure I can continue doing quarantine again although there are some reasons that holding me back	4.114
When I start not to go outside to do activities outside the quarantine place, I'm sure I can continue it even though some time ago there was a need that made me have to go out	4.088
I believe that I can start again to keep my distance or separate myself from family members, although I have been tempted to break it	4.280
I mean it, that from now on I can start to use mask again all the time whenever I meet other people, even though I have taken it off	4.526
I have ever forgotten to monitor daily symptoms, such as body temperature, fever, and cough; however, I'm sure I can keep doing it	3.958
**Behavior**	
I don't go out to do activities outside the quarantine area	3.600
I separate myself or keep my distance from other people	4.410
I wear a mask all the time when there are other people in the room	4.485
I cover my nose and mouth when I sneeze or cough	4.720
I don't share personal tools with others	4.297
I clean the surface of the things I touch	4.266
I wash my hands with soap and running water or hand sanitizer	4.549
I do daily monitoring such as body temperature and cough and cold symptoms	4.037
